# *Lactobacillus fermentum* AGR1487 cell surface structures and supernatant increase paracellular permeability through different pathways

**DOI:** 10.1002/mbo3.260

**Published:** 2015-05-06

**Authors:** Ranjita Sengupta, Rachel C Anderson, Eric Altermann, Warren C McNabb, Siva Ganesh, Kelly M Armstrong, Paul J Moughan, Nicole C Roy

**Affiliations:** 1 Food Nutrition & Health Team, Food & Bio-based Products Group, AgResearch GrasslandsPalmerston North, 4442, New Zealand; 2 Riddet Institute, Massey UniversityPalmerston North, 4442, New Zealand; 3 Rumen Microbiology Team, Animal Nutrition & Health Group, AgResearch GrasslandsPalmerston North, 4442, New Zealand; 4 AgResearch GrasslandsPalmerston North, 4442, New Zealand; 5 Bioinformatics Mathematics & Statistics Team, AgResearch GrasslandsPalmerston North, 4442, New Zealand; 6 Gravida: National Centre for Growth and Development, The University of AucklandAuckland, New Zealand

**Keywords:** germ-free rodents, intestinal barrier integrity, Inflammatory, *Lactobacillus fermentum*, tight junctions

## Abstract

*Lactobacillus fermentum* is commonly found in food products, and some strains are known to have beneficial effects on human health. However, our previous research indicated that *L. fermentum* AGR1487 decreases in vitro intestinal barrier integrity. The hypothesis was that cell surface structures of AGR1487 are responsible for the observed in vitro effect. AGR1487 was compared to another human oral *L. fermentum* strain, AGR1485, which does not cause the same effect. The examination of phenotypic traits associated with the composition of cell surface structures showed that compared to AGR1485, AGR1487 had a smaller genome, utilized different sugars, and had greater tolerance to acid and bile. The effect of the two strains on intestinal barrier integrity was determined using two independent measures of paracellular permeability of the intestinal epithelial Caco-2 cell line. The transepithelial electrical resistance (TEER) assay specifically measures ion permeability, whereas the mannitol flux assay measures the passage of uncharged molecules. Both live and UV-inactivated AGR1487 decreased TEER across Caco-2 cells implicating the cell surfaces structures in the effect. However, only live AGR1487, and not UV-inactivated AGR1487, increased the rate of passage of mannitol, implying that a secreted component(s) is responsible for this effect. These differences in barrier integrity results are likely due to the TEER and mannitol flux assays measuring different characteristics of the epithelial barrier, and therefore imply that there are multiple mechanisms involved in the effect of AGR1487 on barrier integrity.

## Introduction

Lactobacilli are an important part of the human intestinal microbiota, considered to be protective organisms contributing to the maintenance of gastrointestinal tract (GIT) function. Many *Lactobacillus* species have been used as probiotics in foods and nutraceuticals. There is accumulating evidence of the effectiveness of probiotics in reducing the incidence and symptoms of intestinal diseases, including rotavirus infection (Szymanski et al. 2006), antibiotic-associated diarrhea (Vanderhoof et al. 1999), and irritable bowel syndrome (Kim et al. 2005). However, studies have shown that different strains within the same species of lactobacilli can evoke different responses in the host and, therefore, results from one strain cannot be generalized to others (Jacobsen et al. 1999; Christensen et al. 2002; Mackenzie et al. 2010).

Lactobacilli benefit the host through a wide variety of mechanisms that are species and strain specific. These mechanisms include protecting the host from pathogen invasion by competitive exclusion, interacting with the epithelium to modulate the immune response of the host and preserving barrier integrity of the GIT (reviewed by Lebeer et al. 2010, Lebeer et al. 2010). These effects of lactobacilli on the host can be mediated by the cell surface structures of the bacterium (Sengupta et al. 2013) and/or secreted metabolites (Ross et al. 2010).

*Lactobacillus fermentum*, which is commonly found in food products, is considered a “generally recognised as safe” (GRAS) organism by the US Food and Drug Administration and is on the European Food Safety Authority’s list of biological agents with a “qualified presumption of safety” (QPS) (EFSA 2011). Clinical studies have reported that some strains of *L. fermentum* have beneficial effects on human health. For example, *L. fermentum* CECT5716, a probiotic strain isolated from human breast milk, reduces the incidence of upper respiratory tract and GIT infections in infants (Maldonado et al. 2012). However, our previous research has indicated that other *L. fermentum* strains may decrease intestinal barrier integrity (Anderson et al. 2010), and thus potentially may adversely affect human health.

Human oral isolate *L. fermentum* AGR1487 reduces in vitro measures of intestinal barrier integrity (Anderson et al. 2013). It decreases transepithelial electric resistance (TEER) and increases mannitol flux across layers of intestinal epithelial cells, both of which indicate an increase in paracellular permeability. Intestinal epithelial cells co-cultured with AGR1487 had an increase in tubulin gene expression and abundance of microtubule proteins, which has been linked to the disassembly of tight junctions (Yap et al. 1995). This previous research also showed that AGR1487 supernatant does not cause a decrease in TEER, implicating cell surface structures in bringing about this effect.

The hypothesis of this research was that cell surface structures of AGR1487 are responsible for the observed negative effect on intestinal barrier function. AGR1487 was compared to another human oral *L. fermentum* strain, AGR1485, which does not affect TEER (Anderson et al. 2013). Genome size and phenotypic traits associated with the composition of cell surface structures (i.e., sugar utilization capability, acid tolerance, and bile tolerance) were examined, and the effect of bacterial growth phase on TEER was determined. Supernatants produced by bacteria alone and by the bacteria in response to the presence of live intestinal epithelial cells, intestinal epithelial cell debris, and intestinal epithelial cell supernatant, were tested to investigate whether the previous lack of effect of AGR1487 supernatant on TEER (Anderson et al. 2013) was due to an absence of stimulus by intestinal epithelial cells. Finally, the effects of live versus inactivated bacteria were compared to determine whether cell surface structures alone cause a reduction in TEER and an increase in mannitol flux across the cell layer.

## Materials and Methods

### Bacterial strains and cell culture

*Lactobacillus fermentum* strains AGR1485 and AGR1487 were originally obtained from the oral cavity of human adult volunteers as previously described (Anderson et al. 2010). The volunteers gave written consent, and ethical approval from the New Zealand Health and Disability Committee was not required as the sampling was non-invasive. AGR1485 was isolated from a healthy individual and AGR1487 was isolated from an apparently healthy individual who was later diagnosed with ulcerative colitis. The two strains were grown from frozen stocks (glycerol stocks stored at −80°C) on de Man, Rogosa, and Sharpe (MRS) agar plates for 48 h at 37°C in 5% CO_2_, then inoculated into 10 mL of MRS broth and grown overnight at 37°C in 5% CO_2_.

Stock cultures of the human colorectal adenocarcinoma Caco-2 cell line (ATCC HTB-37, Manassas, VA, USA ) were grown in T75 flasks in Medium 199 (M199; GIBCO, Invitrogen Corporation) supplemented with 10% fetal bovine serum (FBS; GIBCO, Invitrogen Corporation, Auckland, NZ), 1% non-essential amino acids (NEAA; MEM non-essential amino acids 100× solution; Sigma-Aldrich) and 1% penicillin-streptomycin (PenStrep: 10,000 units/mL penicillin G sodium salt and 10,000 *μ*g/mL streptomycin sulfate in 0.85% saline; GIBCO, Invitrogen Corporation, Auckland, NZ) at 37°C in 5% CO_2_. The medium was replaced every 3–4 days and the cells were subcultured weekly at a ratio of 1:3.

### Pulsed-field gel electrophoresis (PFGE)

PFGE was carried out as described previously (Kelly et al. 2010). Briefly, microbial cells were embedded in 2% pulsed-field-certified low-melt agarose (Bio-Rad, Hercules, CA) and subsequently lysed with 10 mg/mL lysozyme. Slices of the agarose plugs (1–3 mm) were digested with restriction enzyme I-Ceu I according to the manufacturer’s instructions. The digested plugs were loaded onto a 1% agarose gel (Seakem Gold Agarose). The gel was run for 20 h, in 0.5× TBE at 14°C using a CHEF-DR® III system powered by a Powerpac Basic (Bio-Rad). The gel was subsequently stained with ethidium bromide for 30 min and bands were visualized and captured using a Kodak Gel Doc Box (KODAK Gel Logic 200 Imaging system) modified to accommodate a remotely controlled (Nikon Camera Control Pro 2) Nikon D700 digital camera (Nikon Corporation, Tokyo, Japan). The raw images were developed in Adobe Photoshop Lightroom 2.5.

### Sugar utilization

The sugar utilization capabilities of the two *L. fermentum* strains were compared because differences could impact on the composition of the bacterial cell surface structures. The API50CH carbohydrate metabolism kit (bioMérieux, Craponne, France) was used as described by the manufacturer. Briefly, an aliquot (1.5 mL) of overnight broth from each strain was centrifuged and resuspended in CHL medium (bioMérieux) to an optical density at 600 nm (OD_600nm_) of 0.451. The API50CH strips were filled with the inoculated medium and incubated at 37°C for 48 h. Sugar utilization patterns were recorded for each strain tested. Strain identification via API 50CH was conducted using the api*web*™ identification software v5.1 (https://apiweb.biomerieux.com).

### Bile and acid tolerance

The ability for the two *L. fermentum* strains to survive in the presence of bile and acid was assessed to determine whether these oral isolates were likely to survive passage through the stomach and colonize the GIT. To test the effect of bile on viability of the two *L. fermentum* strains, broth (Walker and Gilliland 1993) and agar assays (Kaboré et al. 2012) were carried out. For broth assays, triplicates of aliquots (10 mL) of control MRS broth and MRS broth supplemented with 0.3% bile (Sigma-Aldrich, Auckland, NZ) were inoculated with 100 *μ*L of AGR1485 or AGR1487 overnight culture and incubated at 37°C. OD_600nm_ was measured hourly for 6 h. For the agar assay, eight 10-fold serial dilutions of an overnight culture (20 *μ*L) were applied onto agar plates supplemented with increasing concentrations of bile (0%, 0.5%, 1%, 1.5%, and 2%). The concentrations of bile were chosen to represent the range found throughout the human GIT. Colonies were counted after 48 h of incubation at 37°C in 5% CO_2._

To test the effect of acidic conditions on the viability of the two *L. fermentum* strains, overnight cultures were centrifuged (5000 *g* for 5 min) and pellets resuspended in MRS broth (control), and MRS broth acidified to pH 4 and pH 2 (pH adjusted with 6 mol/L HCl) to mimicking the pH range found in the human stomach. At 0, 2, and 4 h aliquots were removed, serial diluted, and applied on agar plates for enumeration as described above. The time points (2 and 4 h) were chosen to represent the time it takes for food to pass through the human stomach to the small intestine.

### Transepithelial electrical (TEER) assay

The TEER assay was used to measure the effect of treatments on the integrity of the tight junctions between Caco-2 cells as a model of human intestinal epithelium (le Ferrec et al. 2001; Shah et al. 2006). Caco-2 cells were seeded in 12 mm polyester Transwells with 0.4 *μ*m membrane pore size (Corning Incorporated, Corning, NY, USA) in 12-well plates at a concentration of 2 × 10^5^ cells/Transwell. Cells were grown in M199 supplemented with 10% FBS, 1% NEAA, and 1% PenStrep at 37°C in 5% CO_2_ for 15–19 days until they formed a differentiated monolayer (le Ferrec et al. 2001). About 24 h prior to the TEER assay, medium from the Caco-2 cell monolayers was replaced with M199 supplemented with 1% NEAA. FBS was not included because its presence leads to high production of lactic acid by the bacteria, which in turn negatively affects the Caco-2 cells. PenStrep was not included because it would limit the bacteria viability. The initial resistance values of Caco-2 monolayers were recorded using an electrode chamber (ENDOHM-12 tissue culture chamber; World Precision Instruments, Sarasota, FL, USA) and voltohmeter (EVOM Epithelial Tissue Voltohmmeter; World Precision Instruments), and the TEER values were calculated using equation 1. Caco-2 monolayers that had an initial TEER greater than 500 ohms/cm^2^ were used for assays. The monolayers were assigned to treatment groups so that all treatment groups had a similar mean initial TEER and similar variation within the group.

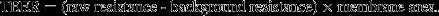
1

Calculation of TEER from raw resistance values. Background resistance was measured to be 12 ohms and the membrane area was 1.12 cm^2^.

After the initial TEER measurements were taken, the apical medium from the Transwells was replaced with the treatment solutions. In all assays, control medium (M199 supplemented with 1% NEAA), live AGR1485 (OD_600nm_ 0.9), and live AGR1487 (OD_600nm_ 0.9) were included, as well as treatments described in the following sections. An OD_600nm_ 0.9 equates to approximately 10^9^ CFU/mL. Three independent assays each with four replicates per treatment were carried out (total *n* = 12 per treatment). TEER was measured every 2 h for 12 h and the change in TEER at each time point was calculated using equation 2.

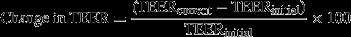
2

Calculation of change in TEER from TEER values. TEER_initial_ was the TEER at time zero before the treatments were added.

### Effect of bacterial growth phase on TEER

Bacteria from the same culture harvested at six different phases of their growth were used as treatments in the TEER assay. A 100 *μ*L aliquot of each strain was inoculated into 10 mL MRS broth (three tubes/strain) and incubated at 37°C in 5% CO_2_. The OD_600nm_ was measured every 2 h for 24 h and was plotted against time. The OD_600nm_ corresponding to the early and late log phases for each strain was determined by linear regressions using Sigma Plot 10 (Systat Software, San Jose, CA, USA), and the mid log OD_600nm_ was calculated as the midpoint between the early and late log phases. The OD_600nm_ corresponding to transition and early log phase was manually chosen based on the shape of the growth curve. The late stationary phase was selected on the basis of the time (18 h after inoculation used in previous experiments, Anderson et al. 2010) to maintain consistency. Bacterial cells were collected at the determined phases of their growth when the inoculated culture reached the corresponding OD_600nm_ or time point. Bacterial cells were centrifuged (5000 *g* for 5 min), the cell pellet was washed once in M199 supplemented with 1% NEAA and resuspended in the same medium to an OD_600nm_ of 0.9 ensuring a constant number of bacterial cells from each growth phase.

### Effect of bacterial supernatant on TEER

**Figure 1:**
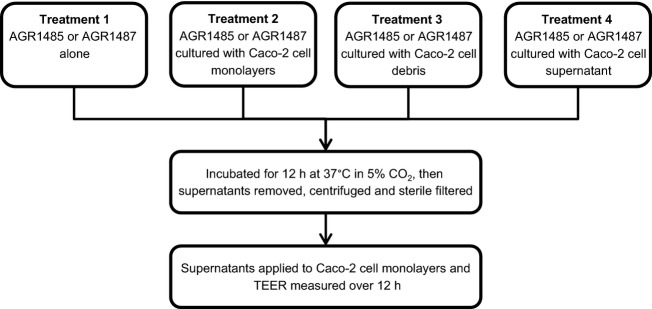
Four treatment groups of AGR1485 and AGR1487 supernatants that were produced for testing in the TEER assay. All treatments were made in M199 supplemented with 1% nonessential amino acids.

In order to test whether the bacteria required stimulation from intestinal epithelial cells to produce metabolites that alter TEER four different bacterial supernatant treatments were prepared for each *L. fermentum* strain: (1) bacteria alone; (2) bacteria cultured with live Caco-2 cell monolayers; (3) bacteria cultured with Caco-2 cell debris; and (4) bacteria cultured with Caco-2 cell supernatant (Fig.1). For all treatments, the bacteria were added at OD_600nm_ 0.9 and incubated for 12 h at 37°C in 5% CO_2_ to allow metabolites to be secreted. The cell debris used in Treatment 3 was generated from Caco-2 cell monolayers homogenized using an OMNI-TH homogenizer (OMNI International, Tulsa, OK, USA) at the highest speed setting for 30 sec. The cell disruption was verified under the microscope using trypan blue. For Treatment 4, Caco-2 cell monolayers were grown in M199 supplemented with 1% NEAA for 12 h, and then the Caco-2 cell supernatant was collected, centrifuged (5000 *g* for 5 min), and filter sterilized (0.22 *μ*m filter). After the 12 h treatments the supernatants were then removed, centrifuged, and sterile filtered prior to addition to the Caco-2 cell monolayers for the TEER assay.

### Effect of UV-inactivated bacteria on TEER

The two *L. fermentum* strains were inactivated using UV treatment prior to testing in the TEER assay. Overnight bacterial culture (1 mL) was centrifuged (5000 *g* for 5 min) and the bacterial cell pellets were resuspended in M199 supplemented with 1% NEAA (OD_600nm_ 0.9). The resuspended bacteria (2 mL) were added to each well of a 6-well microtitre plate. The plate was placed on ice below a UV lamp (UVP 3UV-38, Bio-Strategy LTD) and exposed to UVC radiation (254 nm) for 30 min to achieve 100% inactivation for both strains. The UV-treated bacteria were inoculated onto MRS agar plates and incubated at 37°C in 5% CO_2_ to ensure no viable bacteria remained.

### Effect of UV-inactivated bacteria on mannitol flux

Caco-2 cell layers, live bacteria treatments and UV-inactivated bacteria were prepared as described above. For every 2 mL of treatment (500 *μ*L/Transwell), 80 *μ*L of ^3^H-mannitol stock solution (^3^H-mannitol in 90% ethanol; American Radiolabelled Chemicals, St. Louis, MO) was added to a final concentration of 9.25 × 10^4^ Bq/mL. Three separate experiments were carried out with four replicates per treatment for each experiment (total *n* = 12 per treatment). During the experiment, 75 *μ*L of cell culture medium was removed from each well every 2 h for 12 h. All samples were mixed with scintillation fluid (StarScint, Perkin Elmer, Waltham, MA) at a ratio of 1:1 and counted using a 1459 Microbeta Trilux scintillation counter (Perkin Elmer). The fluorescence detected from ^3^H was used to calculate the amount of ^3^H-mannitol in each well at each time point using equation 3 and the percentage of mannitol that had passed across the cell layer was calculated using equation 4.

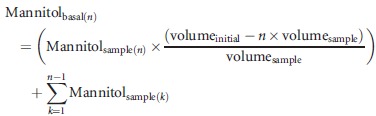
3

Calculation of the amount of ^3^H-mannitol present in each basal well (Mannitol_basal(*n*)_) at each time point (*n*). Mannitol_sample(*n*)_ was the disintegrations per minute (dpm) for the sample taken at time point *n*. Mannitol_basal(*n*)_ was the amount of ^3^H-mannitol in the basal well at time point *n*, taking into consideration the initial volume of the basal well (volume_initial_ = 1500 *μ*L), volume of sample removed (volume_sample_ = 75 *μ*L), and the amounts removed for sampling at time points previous to *n* (*k* = 1 to *n* − 1).

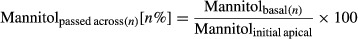
4

Calculation of the percentage of ^3^H-mannitol passed across the cell layer to the basal compartment, taking into consideration the amount of ^3^H-mannitol that was initially added to the apical compartment.

### Statistical analyses

For the broth bile tolerance test, acid tolerance test, and mannitol flux assay a three-way repeated measures analysis of variance (ANOVA) test was applied to the data to test the effects of strain, treatment, time, and their interactions. For the bile agar test a two-way ANOVA was used to analyze the effects of strain, treatment, and their interactions, and the *P*-value was adjusted by the Benjamini Hochberg method (Benjamini and Hochberg 1995) for controlling the false discovery rate. A log10 transformation was applied to the data from the bile agar test, acid tolerance test, and mannitol flux assay to satisfy the validation of ANOVA assumptions (assuming homogeneity of variance and normal distribution). Statistical analyses were done using the R software (RCoreTeam 2014) and treatments were considered statistically different when *P* < 0.05.

TEER treatments were compared using residual maximum likelihood (REML) analysis with an unstructured covariance model to take account for the repeated measures using the R software (RCoreTeam), including packages “nlme” and ”predictmeans.” Treatments were considered statistically different when the difference between the means at a given time point was greater than the least significant difference level at 5% probability (5% LSD).

## Results

### AGR1487 had a smaller genome than AGR1485

**Figure 2:**
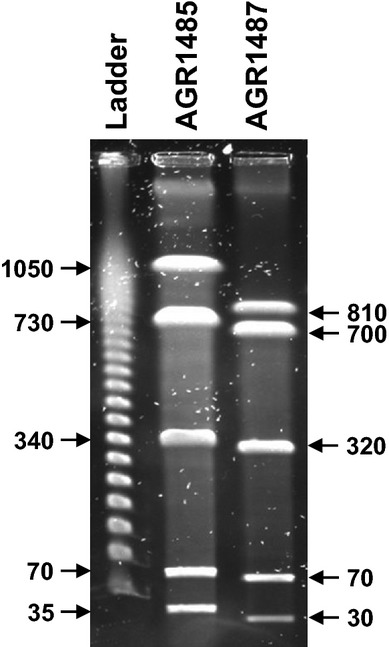
Pulsed-field gel electrophoresis of AGR1485 and AGR1487 genomic DNA digested with restriction enzyme I-CeuI. The marker ladder contained lambda DNA, where the fragments were multimers of 48.5 kb (from gel bottom upwards 48.5, 87, 145.5, 194 kb etc.). The values given are the sizes (kb) of the DNA fragments from the bacterial strains.

There were differences between the PFGE band patterns of the genomic DNA for the two *L. fermentum* strains after digestion with the restriction enzymes I-CeuI (Fig.2). In particular, there were three deletions in the AGR1487 genome compared to the AGR1485 genome (320 kb vs. 340 kb, 700 kb vs. 730 kb, and 810 kb vs. 1050 kb). Comparison of the fragment sizes generated as a result of I-CeuI digests indicated that the genome size of AGR1485 was approximately 2225 kb while that of AGR1487 was approximately 1930 kb.

### Differences between sugar utilization patterns in AGR1485 and AGR1487

As expected, the sugar utilization profiles of AGR1485 and AGR1487 matched those expected for the species *L. fermentum* in the apiweb™ identification software. However, the strains differed in their respective range of fermentable sugars. Of the 49 sugars tested, both strains were able to utilize d-ribose, d-galactose, d-glucose, d-fructose, esculin ferric citrate, d-maltose, d-melibiose, d-saccharose, and d-raffinose. AGR1485, but not AGR1487, was also able to utilize d-lactose, d-mannose, and potassium gluconate; whereas AGR1487, but not AGR1485, was able to utilize l-arabinose, d-xylose, and d-trehalose.

### AGR1487 was more tolerant to the presence of bile and acidic conditions than AGR1485

**Figure 3:**
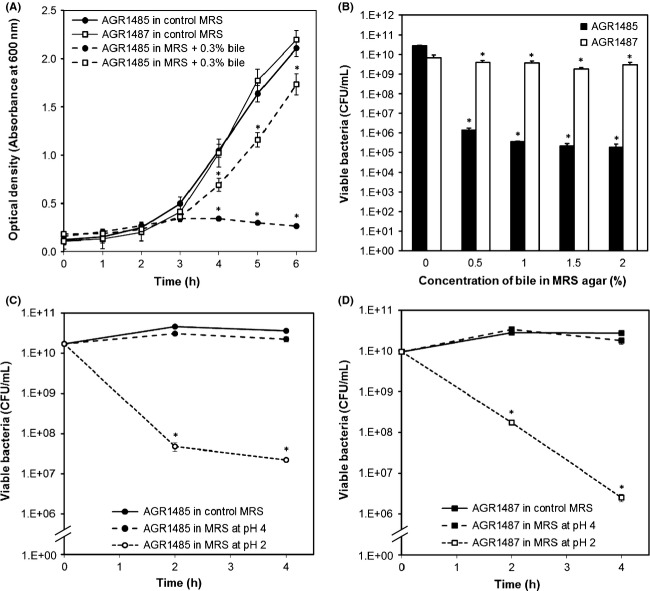
Viability of AGR1485 and AGR1487 in (A) 0.3% bile in MRS broth, (B) different concentrations of bile in MRS agar, and (C and D) MRS broth at different pH values. The values plotted are the means of three replicates and the error bars show SEM. *P < 0.05 compared to control MRS broth or agar at the same time point.

The growth of AGR1485 was inhibited by the addition of 0.3% bile to MRS broth, resulting in an 88% reduction in OD_600nm_ compared to the control (0% bile) after 6 h (Fig.3A). In contrast, AGR1487 exhibited only a 17% reduction in OD_600nm_ after 6 h in the presence of 0.3% bile. Similarly, when inoculated on MRS agar supplemented with bile, the viability of AGR1485 decreased by 4.5 log units in the presence of 0.5% bile and 5.5 log units in the presence of 2% bile; whereas, AGR1487 lost less than 1 log unit of its viability at 2% bile supplementation (Fig.3B).

Both strains were able to tolerate moderately acidic conditions (pH 4 for 4 h) without any significant loss in cell viability compared to that in control medium (Fig.3C and D). At pH 2 the viability of AGR1485 was reduced by 3.5 log units within 2 h, whereas AGR1487 showed a reduced viability of only 2 log units. After 4 h, the viability of both AGR1485 and AGR1487 decreased by 3.5–4 log units.

### Growth phase did not alter the effects of AGR1485 and AGR1487 on TEER

**Figure 4:**
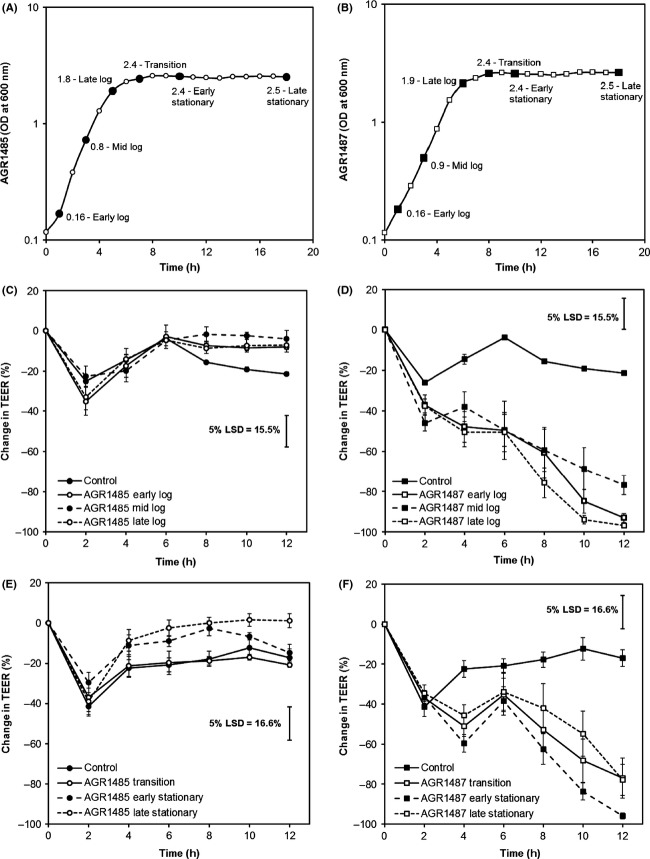
Effect of AGR1485 and AGR1487 at different growth phases on TEER across Caco-2 cell monolayers over time. Growth curves of (A) AGR1485 and (B) AGR1487 showing the optical densities that were selected to represent each growth phase (black data points). Values shown are the means of three replicates. Effect of log phase (C) AGR1485 and (D) AGR1487, and stationary phase (E) AGR1485, and (F) AGR1487 on TEER. The plotted values are the means of 12 replicates (four replicates per run, three independent runs) and the error bars show the SEM. The least significant difference levels at 5% probability (5% LSD) are given.

The growth curves showed that both *L. fermentum* strains grew exponentially for approximately 6 h and then entered stationary phase which continued for the 18 h studied (Fig.4A and B). AGR1485 did not cause any significant change in TEER compared to the control regardless of its growth phase (Fig.4C and E). AGR1487 caused a significant decrease in TEER compared to the control independent of its growth phase (Fig.4D and F), and there were no significant differences between any of the growth phases at any time point.

### AGR1485 and AGR1487 supernatant did not affect TEER

**Figure 5:**
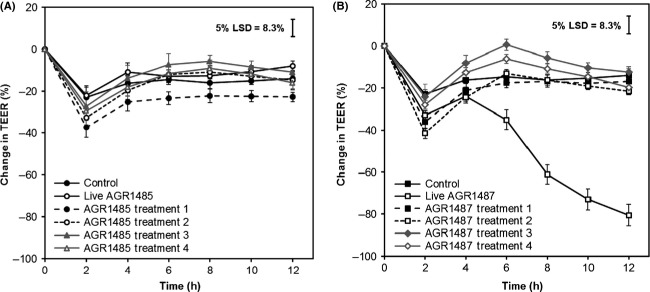
Effect of metabolites produced by (A) AGR1485 or (B) AGR1487 on TEER across Caco-2 cell monolayers over time. Treatment 1: Bacteria alone; Treatment 2: Bacteria cultured with Caco-2 cell monolayers; Treatment 3: Bacteria cultured with Caco-2 cell debris; Treatment 4: Bacteria cultured with Caco-2 cell supernatant. The plotted values are the means of 12 replicates (four replicates per run, three independent runs) and the error bars show the SEM. The least significant difference levels at 5% probability (5% LSD) are given.

Bacterial supernatants were produced alone and in combination with Caco-2 live cells, debris and supernatant (Fig.1). Live AGR1485 and the AGR1485 supernatants did not significantly (*P* > 0.05) alter the TEER compared to the control (Fig.5A). As expected, live AGR1487 caused a decrease in TEER compared to control medium from 6 h onwards (*P* < 0.05); however, none of the four AGR1487 supernatants caused any significant change in TEER compared to the control (Fig.5B), confirming the previously reported finding that the bacterial supernatant does not affect TEER (Anderson et al. 2013).

### UV-inactivation did not alter the effects of AGR1485 and AGR1487 on TEER, but did alter the effect of AGR1487 on mannitol flux

**Figure 6:**
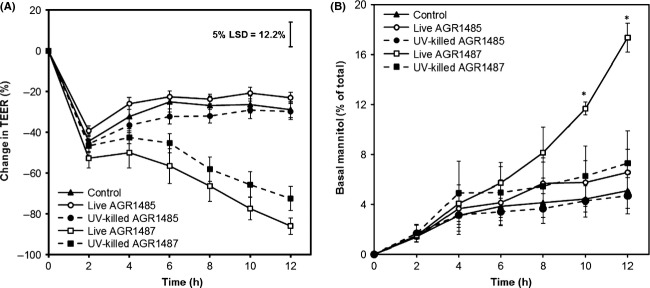
Effect of live and UV-killed AGR1485 and AGR1487 on (A) TEER and (B) mannitol flux across Caco-2 cell monolayers over time. The plotted values are the means of 12 replicates (four replicates per run, three independent runs) and the error bars show the SEM. For the TEER data, the least significant difference levels at 5% probability (5% LSD) is given. For the mannitol flux data, the *indicate *P* < 0.05 compared to the control.

Both live and UV-inactivated AGR1485 did not significantly alter TEER compared to control media; whereas both live and UV-inactivated AGR1487 caused a decrease in TEER (Fig.6A). Pairwise comparison showed no significant difference between the effects of live AGR1487 and UV-inactivated AGR1487 at any time point. For AGR1485, in agreement with the TEER data, the mannitol flux results showed that compared to control medium, neither live nor UV-inactivated AGR1485 changed the rate at which mannitol crossed the Caco-2 cell layers (Fig.6B). As expected, live AGR1487 increased the passage of mannitol across the cell layer compared to control media; 17% versus 5.5% mannitol passed across the cell layer after 12 h (*P* < 0.05). Surprisingly, UV-inactivated AGR1487 did not cause an increase in mannitol flux compared to control medium, and pairwise comparison showed that there were significant differences between the effects of live AGR1487 and UV-killed AGR1487 on the percentage of mannitol that had crossed the cell layer at 10 and 12 h.

## Discussion

The results of this research support the hypothesis that the cell surface structure of AGR1487 is critical for its negative effect on intestinal epithelial barrier function in vitro, but also provide evidence that secreted compounds play a role. Both live and UV-inactivated AGR1487 decreased TEER across Caco-2 cells; however, only live AGR1487, and not UV-inactivated AGR1487, increased the rate of passage of mannitol between the Caco-2 cells. These differing results likely reflect that the TEER and mannitol flux assays measure different characteristics of the epithelial barrier, and imply that there are multiple mechanisms involved in the effect of AGR1487 on barrier integrity.

In terms of the effect on TEER, the data showed that the decrease brought about by live AGR1487 was independent of the growth phase of AGR1487, and molecules secreted by AGR1487 alone and in combination with Caco-2 cells, did not cause the same decrease in TEER. These results imply that an interaction between the bacterium’s cell surface structural components is required to reduce TEER. In contrast, both live and UV-inactivated AGR1485 did not affect the TEER across Caco-2 cell layers, suggesting that the bacterial cell surface components required for these interactions may be absent or poorly expressed in this strain. Interestingly, the PFGE results showed that the genome of AGR1485 is larger than that of AGR1487. Ongoing in-depth analysis of both genome sequences will reveal the presence or absence of genetic functionality that may be associated with the observed effects of the respective phenotypes.

The difference in the physical interaction of the two strains with host cells is supported by previous experiments showing that AGR1487 adheres to Caco-2 cells in greater numbers than AGR1485 (Anderson et al. 2013). These interactions are likely between the bacterial surface components, such as microbe-associated molecular patterns (MAMPs; bacterial cell surface proteins, teichoic acids, lipids, and peptidoglycan), and the epithelial cell surface receptors (e.g., Toll-like receptors, TLR). In support of this, the gene expression of the P38-MAPK signaling pathway is increased in Caco-2 cells treated with AGR1487 compared to those treated with AGR1485 (Anderson et al. 2013). Since MAPK signaling, which leads to tight junction disruption (10), is known to be activated by TLR 2/1 stimulation (8), it implies that AGR1487 caused greater TLR activation than AGR1485.

The difference in cell surface structure may be related to the observed differences in sugar utilizing capabilities between AGR1485 and AGR1487. Studies with *Lactobacillus delbrueckii* subsp. *lactis* 313 suggest that utilization of different sugars stimulate the production of different cell surface proteins (Agyei and Danquah 2012). In turn, variation in the cell surface peptidoglycan composition leads to strain-specific interactions with host cells resulting in differential activation of metabolic pathways and immune responses (Arthur et al. 1992; Macho Fernandez et al. 2011). None of the sugars that are utilized differently by the two strains are present in the cell culture media so it is unlikely that AGR1487 indirectly affects the Caco-2 cells by altering the media composition compared to AGR1485.

The observed difference in tolerance to bile of the two *L. fermentum* strains also implies that they have differences in their cell surface structures, which may in turn affect their interactions with host cells. Lactobacilli encounter various harsh conditions (e.g., bile, low pH, oxidative, and osmotic stress) as they move through the GIT. To survive these conditions, they need to display many resistance mechanisms. For example, genes involved in peptidoglycan and cell surface protein synthesis are known to be differentially expressed after bile exposure (Pfeiler et al. 2007), and teichoic acid structures have been suggested to affect bacterial cell integrity in acidic conditions and in the presence of bile (Wall et al. 2007; Whitehead et al. 2008). The results imply that AGR1487 is better adapted for passage through the GIT; whereas AGR1485 may be more suited to the oral cavity ecosystem or it may have been a transient bacterium present in the mouth due to food consumed by the volunteer from whom it was isolated.

In contrast to the TEER data, the observation that only live but not UV-inactivated AGR1487 increased mannitol flux across the Caco-2 cell monolayers indicates that the effect on mannitol flux may be caused by a compound, or mixture of compounds, produced by the bacterium and not the cell surface structure itself. Although TEER and mannitol flux are both measures of paracellular permeability, the TEER assay specifically measures ion permeability, whereas the mannitol flux assay measures the passage of uncharged molecules. Therefore, it is possible that there are at least two distinct mechanisms involved in the regulation of each of these permeability measures.

The independent regulation of TEER and mannitol flux by different tight junction proteins is a recognized phenomenon, but the mechanism(s) by which this occurs is not fully understood. Tight junctions are highly dynamic structures, involving more than 50 known proteins that are constantly being remodeled due to interactions with external stimuli. The effect of some of the transmembrane proteins on permeability is partially understood. For example, overexpression of the transmembrane protein claudin-4 leads to an increase in TEER but no change in mannitol flux (van Itallie et al. 2001), implying that claudin-4 restricts the paracellular transport of ions, but not uncharged molecules. While, the overexpression of occludin results in increased TEER and increased mannitol flux, implying that occludin allows selective paracellular flux of uncharged molecules in the presence of electrically sealed tight junctions (Balda et al. 1996). However, previous studies have shown that the effect of AGR1487 on Caco-2 cells is not linked to a direct alteration of tight junction expression (Anderson et al. 2013). Instead, Caco-2 cells treated with *L. fermentum* AGR1487 have both a higher expression level of genes encoding for tubulins and a higher abundance of microtubule-associated proteins. A high turnover in tubulin synthesis has been linked to the disassembly of tight junctions (Yap et al. 1995), which indicates that these genes and proteins may play a role in the negative effects on barrier integrity observed for *L. fermentum* AGR1487. How this indirect effect on tight junction integrity relates to the TEER and mannitol permeability measures is currently unknown.

Another possibility is that difference in results between the two barrier integrity measures is related to the size of the molecules not the charge. UV-killed AGR1487 may only be able to reduce the integrity of the tight junctions to less of a degree than live AGR1487, thereby inducing a “gap” that more ions are able to pass through (decreased TEER) but that is too small for more mannitol to pass through (no alteration in mannitol flux).

Overall, these results indicate that AGR1485 and AGR1487 differ in their cell surface structures, and that the surface structure of AGR1487 mediates its effect on decreasing TEER. The changes in the cell envelope may also be responsible for the difference in bile tolerance between the strains. However, the increase in mannitol flux caused by AGR1487 appears to be regulated by an independent mechanism, most likely mediated by compounds secreted by the live bacterium. Future research will focus on identifying the cell surface molecules and secreted compounds responsible for the negative effects of AGR1487 on intestinal epithelial barrier integrity, both by iterative fractionation of the bacterial cell components and by comparing the genome sequences of the two strains. Long-term this research will increase our understanding of host–microbe interactions that affect human intestinal health.
